# Identifying and Overcoming Artifacts in ^1^ H-Based Saturation Transfer NOE NMR Experiments

**DOI:** 10.1021/jacs.2c13087

**Published:** 2023-03-06

**Authors:** J. Tassilo Grün, Jihyun Kim, Sundaresan Jayanthi, Adonis Lupulescu, E̅riks Kupče, Harald Schwalbe, Lucio Frydman

**Affiliations:** † Department of Chemical and Biological Physics, Weizmann Institute of Science, Rehovot 7610001, Israel; ‡ Department of Physics, Indian Institute of Space Science and Technology, Valiamala, Thiruvananthapuram 695547, Kerala, India; § Extreme Light Infrastructure—Nuclear Physics, “Horia Hulubei” National Institute for Physics and Nuclear Engineering, 30 Reactorului Street, 077125 Bucharest-Măgurele, Romania; ∥ Bruker UK Ltd., Welland House, Westwood Business Park, Coventry CV4 9GH, U.K.; ⊥ Institute for Organic Chemistry and Chemical Biology, Center for Biomolecular Magnetic Resonance, Goethe-University, 60438 Frankfurt/Main, Germany

## Abstract

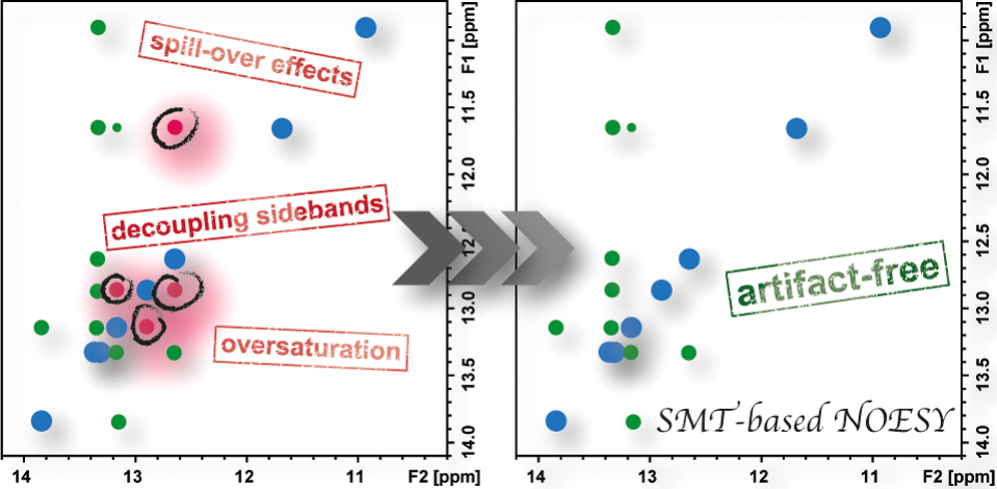

Magnetization transfer experiments are versatile nuclear magnetic resonance (NMR) tools providing site-specific information. We have recently discussed how saturation magnetization transfer (SMT) experiments could leverage repeated repolarizations arising from exchanges between labile and water protons to enhance connectivities revealed via the nuclear Overhauser effect (NOE). Repeated experience with SMT has shown that a number of artifacts may arise in these experiments, which may confound the information being sought – particularly when seeking small NOEs among closely spaced resonances. One of these pertains to what we refer to as “spill-over” effects, originating from the use of long saturation pulses leading to changes in the signals of proximate peaks. A second, related but in fact different effect, derives from what we describe as NOE “oversaturation”, a phenomenon whereby the use of overtly intense RF fields overwhelms the cross-relaxation signature. The origin and ways to avoid these two effects are described. A final source of potential artifact arises in applications where the labile ^1^Hs of interest are bound to ^15^N-labeled heteronuclei. SMT’s long ^1^H saturation times will then be usually implemented while under ^15^N decoupling based on cyclic schemes leading to decoupling sidebands. Although these sidebands usually remain invisible in NMR, they may lead to a very efficient saturation of the main resonance when touched by SMT frequencies. All of these phenomena are herein experimentally demonstrated, and solutions to overcome them are proposed.

## 1 Introduction

Magnetization transfer (MT) NMR experiments have long been used to screen the interaction of low-molecular-weight ligands with biomacromolecules.^1^ MT NMR experiments can also provide site-specific information about biomolecular structures and dynamics. This information can be delivered by either transfer of magnetizations between sites undergoing chemical dynamics in the millisecond regime, as in chemical exchange saturation transfer (CEST),^2−4^ or by spin dynamics between proximate sites, through the nuclear Overhauser enhancement (NOE) effect. 2D NOESY measurements rely on the latter for their operation; NOESY efficiency, however, may suffer when applied to labile NH or OH ^1^H sites, due to losses from chemical exchanges with the aqueous solvent. We have recently proposed a number of ways that can transform this exchange-driven drawback into an advantage, by relying on MT experiments that exploit the replenishments in polarization coming from the solvent water pool.^5−8^ In these experiments, the magnetization at the exchanging site of interest is replenished either by looped excitations and projections,^9,10^ as in loop-projected spectroscopy (L-PROSY),^5^ or by continuous frequency-selective saturation or inversion approaches, like in Hadamard magnetization transfer (HMT),^6^ heteronuclear magnetization transfer,^8^ and selective magnetization transfer (SMT).^7^

All of these experiments aim to probe cross-relaxation; i.e., NOEs from fast exchanging protons in aqueous solution. Hence, they could be particularly useful for the analysis of NOE-connectivities for imino-imino and imino-amino correlations in nucleic acids,^7^ and for elucidating cross-relaxation with amide groups in intrinsically disordered proteins (IDPs).^5^ However, further use of these experiments, and particularly of the SMT approach, can lead to artifacts that need to be considered carefully. We herein describe problems that we have faced on a number of cases, arising from artifacts that can both lead to false apparent NOE cross-peaks, as well as to the suppression of genuine information. Avenues for the identification of these problems and their potential resolution are put forward.

## 2 Results and Discussion

### 2.1 Illustrating the Potential and Perils of Selective Saturation NOE Experiments

**Figure 1:**
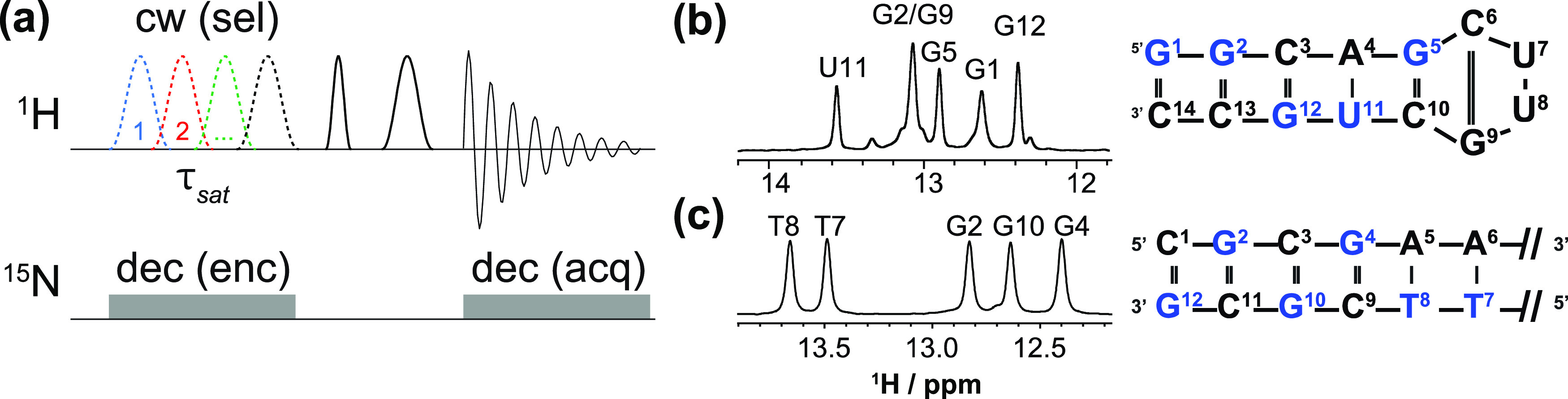
(a) Pulse sequence utilized for the SMT experiment, involving long (800 ms) saturation times that exploit the proton exchanges with water while affecting neighbors by cross-relaxation. If needed, ^15^N decoupling is applied in conjunction with the saturation and acquisition. (b, c) ^1^H spectra of base-paired imino protons arising from a 14-mer hairpin RNA fragment (b, ^15^N-labeled, decoupled) and the palindromic Dickerson dodecamer DNA (c, ^15^N at natural abundance).

Figure 1 illustrates the sequences and systems that this study focuses on. The experiment in question is SMT, an approach that targets the labile peaks of interest one by one with a selective, continuous irradiation. Although devoid of the multiplexing advantages of L-PROSY or HMT, SMT delivers NOE cross-peaks showing an enhanced sensitivity compared to conventional methods, thanks to its reliance on water repolarization. Furthermore, by addressing peaks one by one, SMT was found useful to address processes occurring *within* groups of chemically similar protons, – i.e., imino protons in nucleic acids or amide protons in IDPs. On the other hand, such correlations usually take place in systems that have peculiarities of their own, for instance, close proximity between the chemical shifts of the protons to be correlated, and/or labeling of the bound heteroatoms (e.g., ^15^N) for the sake of subsequent heteronuclear separation.

**Figure 2:**
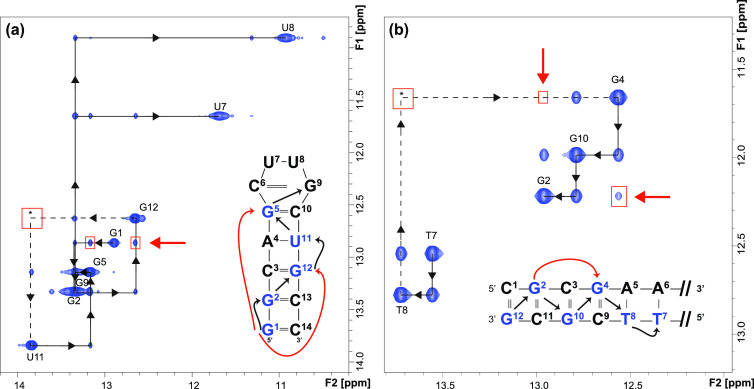
SMT spectrum of (a) a 14-mer RNA (with γB_1_/2π = 10 Hz, 800 ms saturation time, and 500 μs Hz (0.6 W, garp4) ^15^N-decoupling field); and (b) Dickerson’s DNA (with γB_1_/2π = 5 Hz, 800 ms saturation time and no applied ^15^N-decoupling). The expected sequential NOE-connectivities (“sequential walks”) are indicated with black arrows. Dashed lines with asterisk show expected but nonvisible cross-peaks. Unexpected, unreasonable cross-peaks are highlighted in red and by red arrows.

Figure 2 shows the kind of problems that may arise in such experiments. These data, acquired on a 14-mer hairpin RNA and on the palindromic Dickerson dodecamer DNA utilizing SMT and the parameters described in the figure caption, show many of the cross-peaks expected from a quality NOESY experiment. These include G9–U8 correlations in the loop region of the RNA, and G2–G12 correlations in the RNA stem. As discussed previously,^7^ these cross-peaks are magnified several-fold when compared to those in conventional NOESY spectra. As also expected, the enhancement of the various cross-peaks will depend on the solvent exchange rates of the saturated imino protons, hence leading to off-diagonal peak patterns that are typically asymmetric. At the same time, however, the data also show “cross-peaks” (highlighted in red) that correlate the imino proton signals of G1–G5 and G1–G12 for the 14-mer RNA hairpin, and between G2–G4 for the dodecamer DNA. All these correlations are several base pairs apart in sequence, a distance that is unreasonably long.

In general, we have observed two sources of artifacts originating this behavior, as well as one source of artifacts that can suppress genuine cross-relaxation information. Their nature and ways to avoid them are further discussed below.

### 2.2 Overwhelming the NOE: Oversaturation and Spill-Over Effects

**Figure 3:**
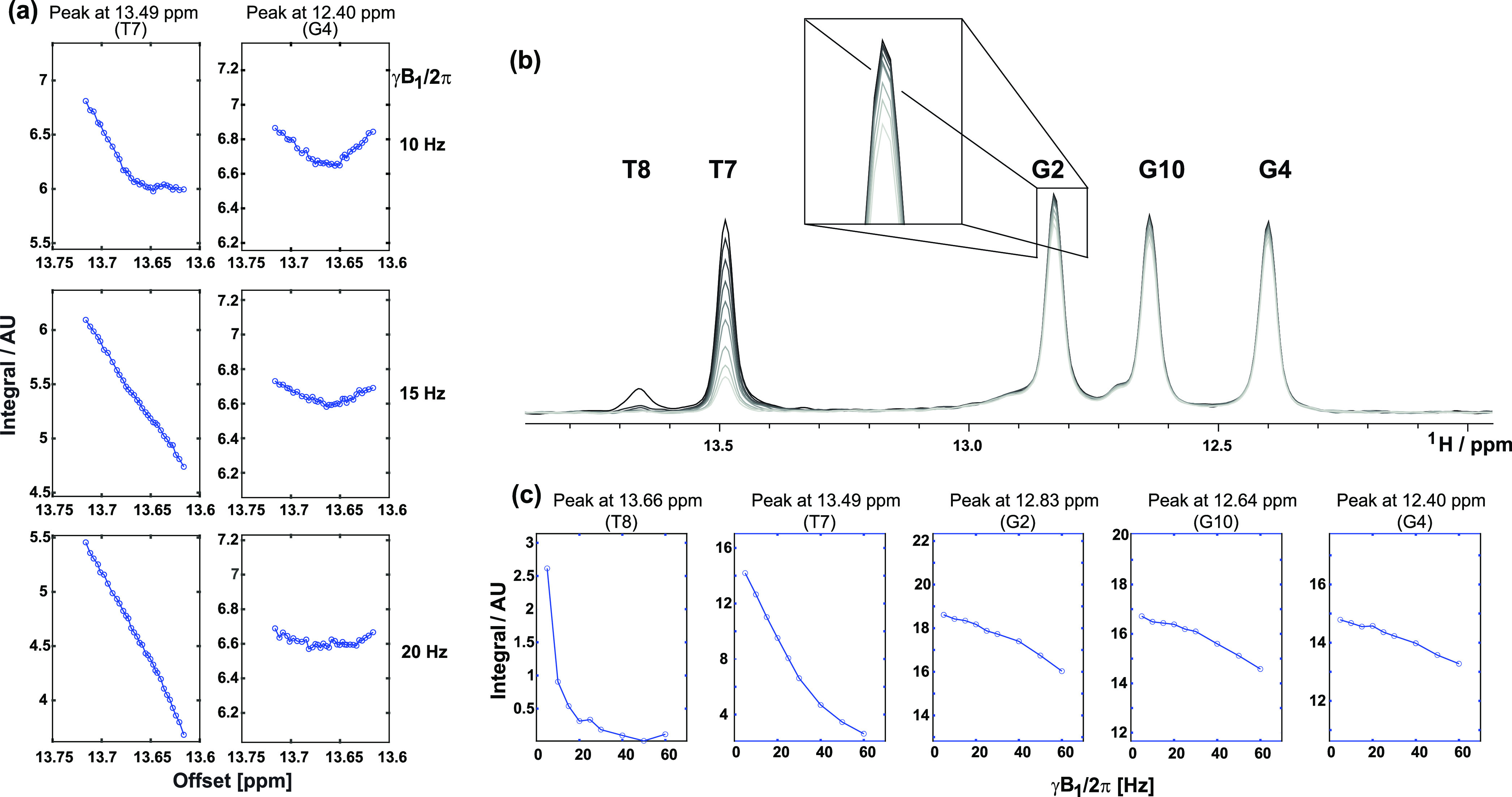
(a) Offset-dependent saturation (T8) and cross-relaxation (G4) profiles with increasing γB_1_ strength (800 ms saturation) observed for the Dickerson DNA. (b, c) Effect of increasing γB_1_ strength (800 ms saturation of T8) on the overall spectra for the DNA, showing in panel (b) an overlay of the 1D spectra and in panel (c) plots of the signal intensities as a function of γB_1_ strength.

To illustrate potential sources of artifacts, we focus first on the dodecamer DNA, which has a well-established structure and a well-dispersed imino proton fingerprint. Starting from a weak (γB_1_/2π = 10 Hz) saturation of the most downfield peak (T8) as a reference point, clear cross-relaxation-induced cross-relaxation effects to the G4 and to the T7 imino sites at 12.40 and 13.49 ppm are observed upon crossing the saturation through the maximum of their neighboring T8 site (Figure 3). These behaviors are characterized by a dip whose depth becomes more pronounced as the frequency of the saturating pulse is varied around the top of the T8 resonance. As the saturation pulse intensity increases from these relatively weak values, however, two confounding effects can be observed. One of these includes the broadening, and eventual smearing out, of the cross-relaxation-induced dips in G4 and T7 (Figure 3a). We refer to this as an “oversaturation” of the NOE information, and its origin can be traced to the increasing line width that both the targeted peak and their cross-relaxing partners will exhibit upon increasing the saturating B_1_. The nature of this effect and guidelines to avoid oversaturation while highlighting NOEs to the maximum possible strength are described in the upcoming paragraph.

In addition to oversaturation, SMT data are deteriorated by a second effect: an increasingly unspecific saturation, causing a partial decrease of all neighboring imino resonances regardless of whether they are cross-relaxing with the targeted ^1^H or not. An example of this “spill-over” effect is shown in Figure 3b,c, which highlights how irradiation of the T8 resonance affects the G2, G10, and even G4 peaks of the DNA. Processing the resulting spectrum by subtraction of an off-resonance saturated spectrum by a T8-irradiated one may then lead to the appearance of a cross-peak (e.g., with G2) related solely to this spill-over effect. Notice that this effect comes on top of the aforementioned oversaturation, and together they may lead to both missing real NOE peaks and to false cross-correlations.

### 2.3 Describing and Avoiding Oversaturation and Spill-Over

To visualize the origin of the aforementioned oversaturation of the cross-relaxation information, we consider the simplest system capable of supporting this effect. This involves two uncoupled spins A and B subject to mutual cross-relaxation and to a radiofrequency (RF) irradiation. Denoting (*X*_A_, *Y*_A_, *Z*_A_) and (*X*_B_, *Y*_B_, *Z*_B_) as the *x*, *y*, and *z* magnetizations of these spins, their evolution will be given by^11^
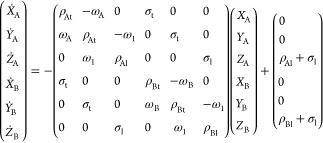
1where ω_A_ and ω_B_ are the chemical shifts of the sites, ω_1_ = γB_1_ is the amplitude of the applied saturating RF, ρ_l_ and ρ_t_ describe the longitudinal and transverse relaxation rates of the two sites, and σ_l_ and σ_t_ are the longitudinal and transverse cross-relaxation rates under the irradiation conditions, respectively. For simplicity, we will assume that the longitudinal and transverse self-relaxation rates are equal for the two sites: ρ_Al_ = ρ_Bl_ = ρ_l_ and ρ_At_ = ρ_Bt_ = ρ_t_. Since SMT’s RF is applied for relatively long times and the chemical shift difference ω_B_ – ω_A_ is relatively large, it is also assumed that the effect of transverse cross-relaxation is minor and hence can be neglected. With these assumptions, the steady-state solution for the longitudinal magnetization components of eq 1 subject to selective irradiation at frequency ω_ir,r_ is
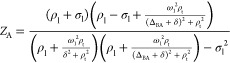
2
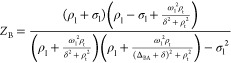
3where Δ_BA_ = ω_B_ – ω_A_ and we have defined the selective irradiation performed near the ω_A_ resonance by an offset δ = ω_A_ – ω_irr_. The validity of eqs 2 and 3 and the neglect of the σ_t_ are justified in Supporting Information 1.

**Figure 4:**
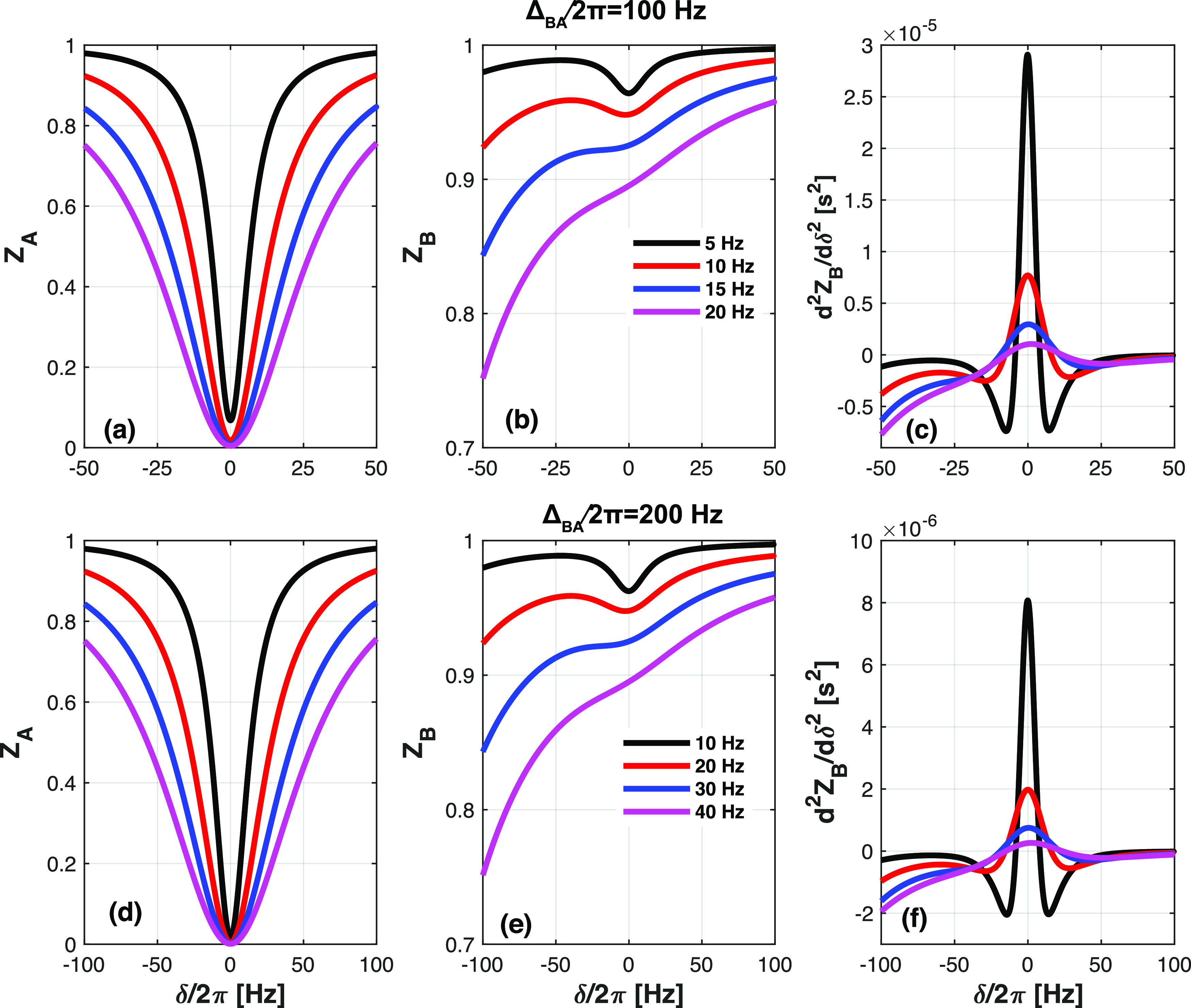
*Z*_A_, *Z*_B_, and *d*^2^*Z*_B_/dδ^2^ as a function of δ/2π for σ_l_ = −0.2 s^–1^, ρ_t_ = 12 s^–1^, and ρ_l_ = 6 s^–1^. Chemical shift differences are (a–c) Δ_BA_/2π = 100 Hz and (d–f) 200 Hz. RF amplitudes are indicated in panels (b) and (e).

Figure 4a,b,d,e displays *Z*_A_ and *Z*_B_ magnetizations derived from eqs 2 and 3 as a function of δ for chemical shift differences Δ_BA_/2π of 100 Hz (a–c) and 200 Hz (d–f), and for a few reasonable RF amplitudes. Notice that, as in the experiments in Figure 3a, these plots predict the disappearance of the NOE-driven “dip” in the *Z*_B_ profile, as the values of RF amplitudes increase. Notice as well the asymmetry in the *Z*_B_(δ) profiles with increasing RF field, as a result of the aforementioned spill-over effects. We find that it is possible to become relatively insensitive to this asymmetric aspect while retaining information about the depth of the NOE dip by focusing on the second derivative *d*^2^*Z*_B_/dδ^2^, which in all instances exhibits a much larger *relative* variation with respect to δ (Figure 4c,f). Notice the simultaneous decrease of both the NOE dip and the (*d*^2^*Z*_B_/dδ^2^)_δ = 0_ value, as the RF amplitude ω_1_ grows. It follows that the value taken by (*d*^2^*Z*_B_/dδ^2^)_δ = 0_ is a good reporter on the feasibility of a given set of conditions for enabling the observation of an intramolecular NOE by selective irradiation: the more positive this value becomes, the easier the observation of intramolecular NOE will be.

Although the exact form of *d*^2^*Z*_B_/dδ^2^ is, even with the aforementioned approximations, too complex to allow easy interpretation (see Supporting Information 2), the dependence of *d*^2^*Z*_B_/dδ^2^ at δ = 0 can be considerably simplified by assuming that ω_1_^2^ is significantly larger than ρ_t_ρ_l_. This is a reasonable assumption for relaxation rates below 10 s^–1^ and RF amplitudes ω_1_/2π ≥ 5 Hz – both common conditions. One can then obtain a description of this reporter
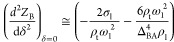
4

The first term in this equation reflects the cross-relaxation effects; as for biomolecules (nucleic acids, polypeptides), σ_*l*_ values are negative, its contribution to (*d*^2^*Z*_B_/dδ^2^)_δ = 0_ will always be positive. This is as desired for an SMT experiment. Notice, however, that its influence decreases quadratically with RF strength ω_1_; this is a manifestation of the aforementioned oversaturation effect. Competing against achieving (*d*^2^*Z*_B_/dδ^2^)_δ = 0_ ≫ 0 is the second term in eq 4, which reflects the spill-over effect. Spill-over clearly carries no cross-relaxation information and makes (*d*^2^*Z*_B_/dδ^2^)_δ = 0_ progressively more negative as ω_1_ increases; its effect, however, decreases as the inverse of the fourth power in the chemical shift difference Δ_BA_ between the correlated peaks. The validity of eq 4 to describe the second derivative is illustrated in Supporting Information 2, Figures S2–S5, for various chemical shift differences and relaxation rates; notice the excellent description offered by eq 4 for SMT-relevant RF ranges.

Equation 4’s simplicity can be used to estimate the largest RF amplitude that will simultaneously be capable of saturating site A while preserving the cross-relaxation effects of site B against spill-over/oversaturation. Indeed, equating eq 4 to zero yields a critical RF amplitude ω_1_^0^
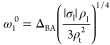
5

**Figure 5:**
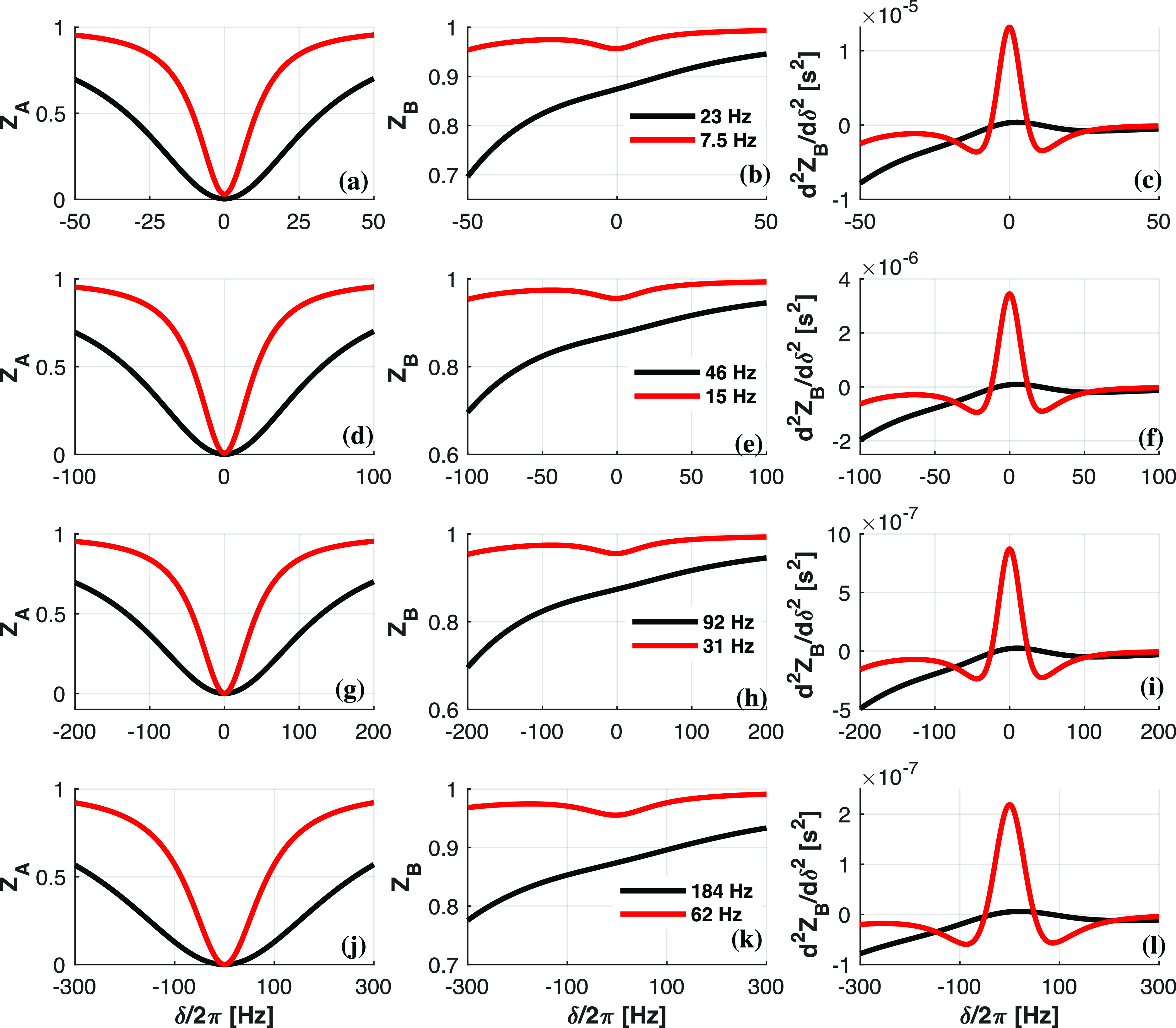
*Z*_A_, *Z*_B_, and *d*^2^*Z*_B_/dδ^2^ as a function of δ/2π and RF amplitudes ≅ ω_1_^0^/2π, . Relaxation rates are σ_l_ = −0.2 s^–1^, ρ_t_ = 12 s^–1^, and ρ_l_ = 6 s^–1^. The chemical shift differences Δ*B*_A_/2π are 100 Hz (a–c), 200 Hz (d–f), 400 Hz (g–i), and 800 Hz (j–l). Notice the different offset ranges utilized for different Δ_BA_.

If the RF amplitude becomes larger than this ω_1_^0^, then (*d*^2^*Z*_B_/dδ^2^)_δ = 0_ will be negative and no NOE will contribute to the *Z*_B_ profile. Decreasing the RF amplitude below this threshold will make (*d*^2^*Z*_B_/dδ^2^)_δ = 0_ > 0, but decreasing it too much will eventually make the NOE difficult to observe. Analyzes of situations involving a range of relaxation rates and chemical shift differences suggest that an optimum RF for making NOE effects large enough is ω_1_^opt^ ≈ ω_1_^0^/3 (Figure 5).

**Figure 6:**
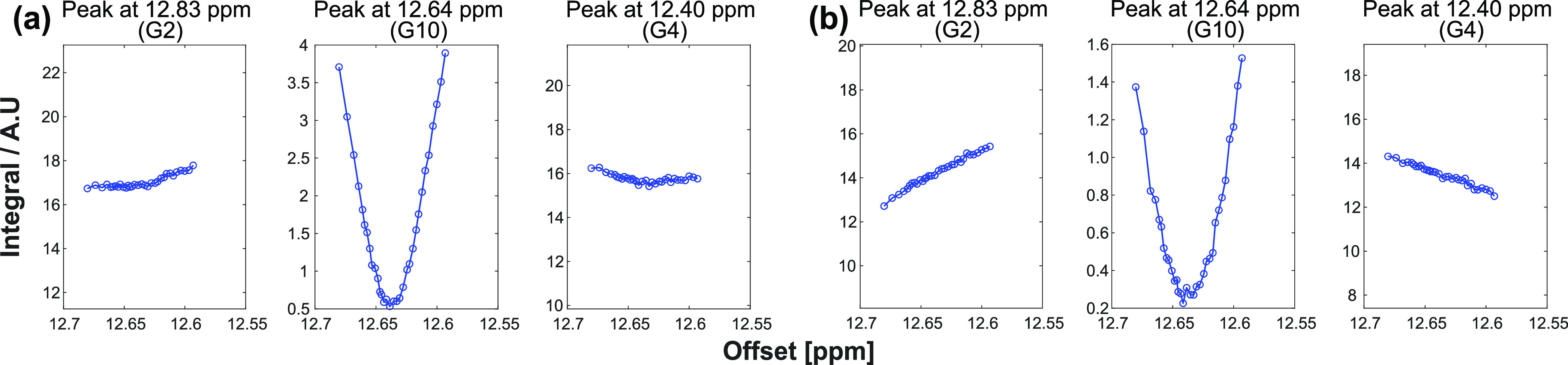
Offset-dependent saturation (G10) and cross-relaxation (G2 and G4) profiles for the Dickerson DNA. While saturation (800 ms) with γB_1_/2π = 10 Hz results in an observable NOE dip (a), a γB_1_/2π = 20 Hz leads to “oversaturation” and the NOE dips are no longer observable (b).

Figure 6 examines these predictions for three closely placed imino resonances in the Dickerson DNA. Taking as a point of reference the saturation of residue G10, a cross-relaxation to both G2 and G4 residues sited ca. 50 Hz apart is to be expected. While the exact values of ρ’s and σ_l_’s for all sites are not known, with average σ_l_ = −0.2, ρ_l_ = 6, and ρ_t_ = 12 s^–1^ rates, eq 5 predicts an ω_1_^opt^/2π ≈ 7 Hz; the profiles in the figure show that while saturation with 10 Hz leads to a weak but detectable NOE peak, saturation profiles at 20 Hz or higher RF fields overwhelm the underlying NOE. Notice that these predictions for the RF values compatible with observations of NOE dips do not imply that SMT will not lead to the sensitivity enhancements that were described in Novakovic et al.;^7^ rather, what they highlight is the need to collect a small number of variable offset experiments with the right RF field strength, when using SMT to ascertain connectivities between nearby resonating sites.

### 2.4 SMT and Heteronuclear Decoupling

**Figure 7:**
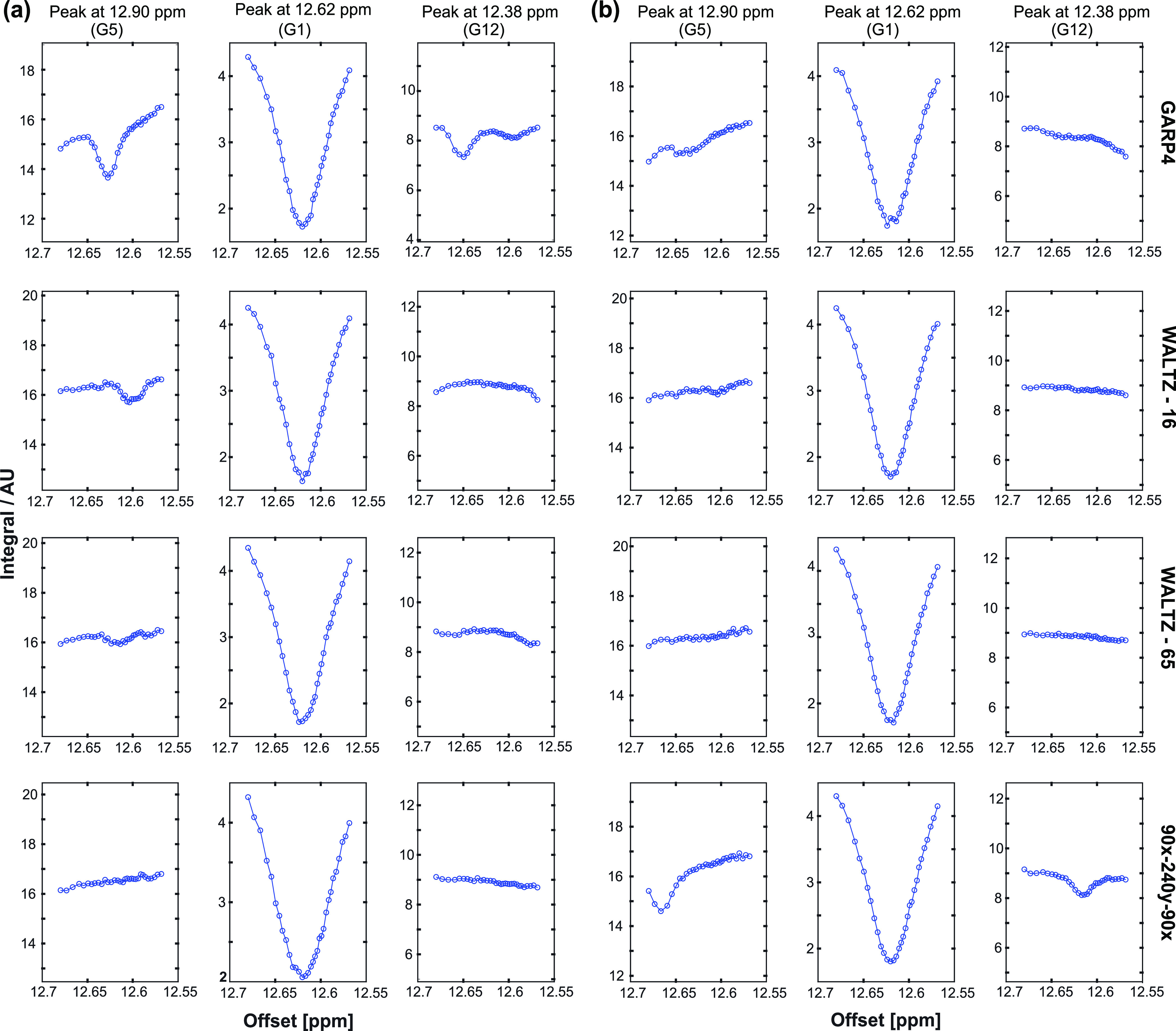
Saturation profiles arising upon continuous saturation (800 ms, 10 Hz) of peak G1 with different ^15^N-decoupling schemes, with τ_90_(^15^N) of (a) 350 μs (1.2 W) and (b) 250 μs (2.4 W). While G1 is saturated, the peak intensities of peaks G5 (left) and G12 (right) are affected at multiples of 1/τ_90_(^15^N)^−1^.

The aforementioned precautions can help to reduce artifacts among closely spaced peaks in SMT experiments. There is, however, another possible source for “false-positive” artifacts, which can lead to the appearance of “cross peaks” – even after variable-offset, low-power experimental precautions have been taken. Moreover, unlike spill-over effects, these additional artifacts can also act among sites that are remarkably far apart (≫γB_1_). Taking residue G1 of the 14-mer RNA hairpin resonating at 12.62 ppm as a case in point, Figure 7 (upper panels) shows the behaviors displayed by the G5 and G12 resonances, positioned +140 and −120 Hz away from G1, as a function of a variable-offset saturation of the latter residue. These peaks show a minor spill-over profile at the γB_1_/2π = 10 Hz used; however, they clearly evidence what at first sight looks like NOE-induced dips on top of these background profiles. Given that the distance in the RNA molecule between the G1 iminos and the G5 and G12 protons is ≈13 and ≈8.5 Å, respectively, these dips must be unrelated to cross-relaxation. Further, on closer look, the maximal dips in these troughs do not align exactly with the maximum expected from the chemical shift of the saturated site. Also, by contrast to what is shown in Figure 3a, the modulation depths of these dips barely varied upon changing the saturation pulse’s intensity.

**Figure 8:**
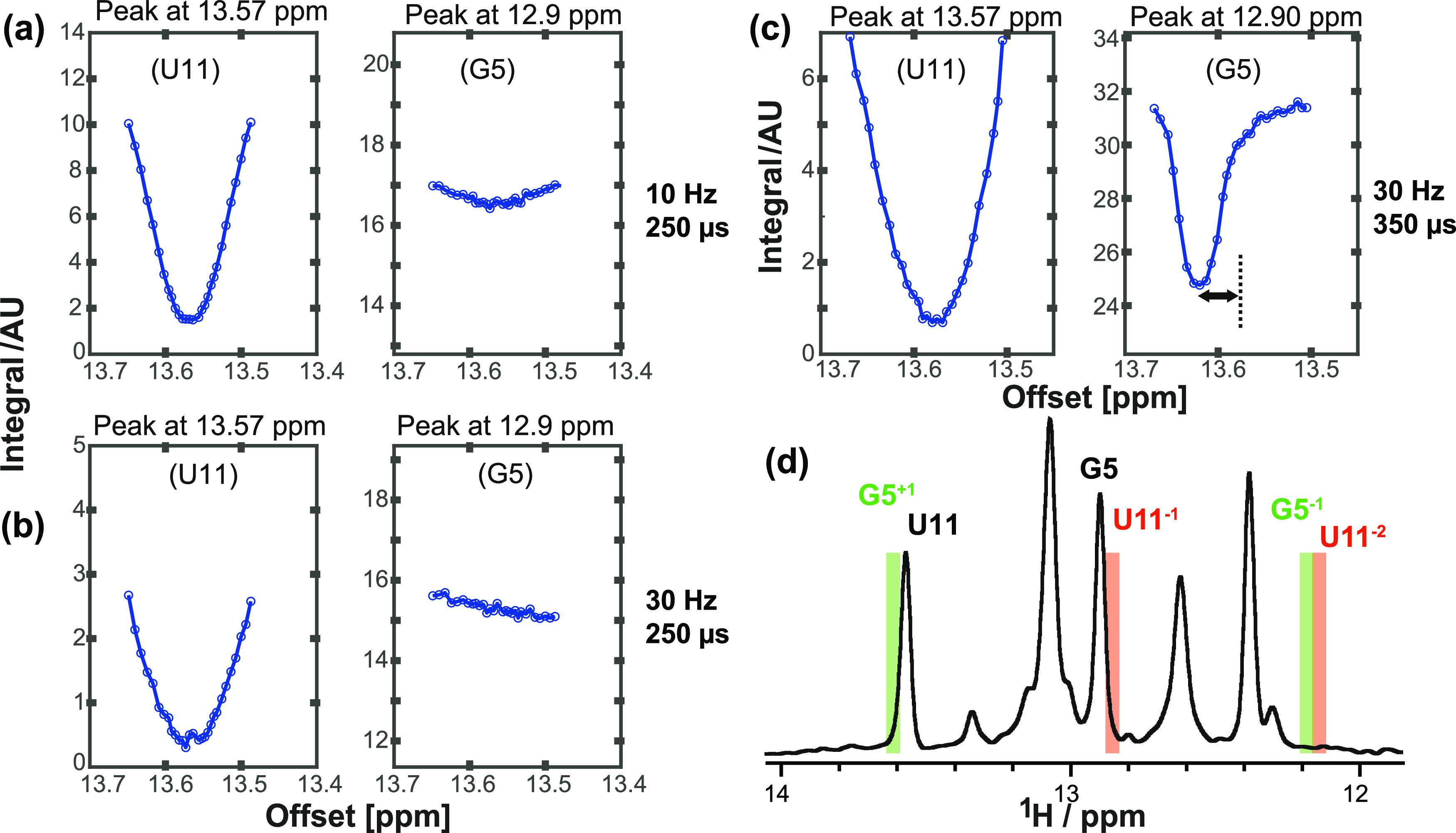
(a–c) Offset-dependent saturation (U11) and cross-relaxation (G5) profiles for the 14-mer RNA. Decoupling power was set to 2.4 W (τ_90_(^15^N) = 250 μs) or 1.2 W (τ_90_(^15^N) = 350 μs) respectively (800 ms saturation); γB_1_/2π strengths are indicated. (c) The assumed “NOE dip” maximum is shifted from the expected chemical shift of the saturated resonance (U11 = 13.6 ppm). (d) Visualization of expected decoupling sidebands for a 90–180–90 decoupling scheme with τ_90_(^15^N) = 350 μs. The expected first and second upfield (−1, −2) and downfield (+1) sidebands are shown in red (U11) and green (G5).

Dips in these experiments are not related to cross-relaxation effects, but rather to the performance of ^15^N decoupling over the course of SMT experiments on ^15^N-labeled samples. Indeed, variations in the decoupling schemes and power (Figure 7) clearly modulate these dips and shift their positions – always in synchrony with multiples of the inverse of the decoupling supercycling time. Figure 8 emphasizes this phenomenon, by showing the saturation behavior of the RNA’s residue U11, which in this hairpin is at a close distance to G5. Applying a 90–180–90 decoupling scheme^14^ with a τ_90_(^15^N) = 250 μs over the course of the saturating pulse leads to the expected NOE with neighboring residue G5 when using γB_1_/2π = 10 Hz and a 800 ms long saturation; this is in turn erased by oversaturation when the saturating field is increased to 30 Hz (Figure 8b). However, when applying lower decoupling powers (τ_90_(^15^N) = 350 μs), a dip of much deeper amplitude affects the intensity of G5, giving the impression of a strong NOE between the latter and U11. Careful inspection, however, reveals a slight offset between the exact minimum of the G5 resonance and U11′s precise chemical shift. The dependence on the dip’s position and decoupling scheme identifies this peak as associated with a decoupling sideband of the nonsaturated (in this case, G5) resonance. For the 90–180–90 decoupling scheme used, these are expected at inverses of the complete 360° decoupling cycle, i.e., at [8*τ_90_(^15^N)]^−1^, placing the first sideband (G5^+1^) coincidently close in frequency with U11. This and other decoupling sidebands are not readily visible in the ^1^H NMR spectrum, but they provide an efficient way to self-saturate the main G5 resonance upon being hit with SMT’s RF (Figure 8d).

**Figure 9:**
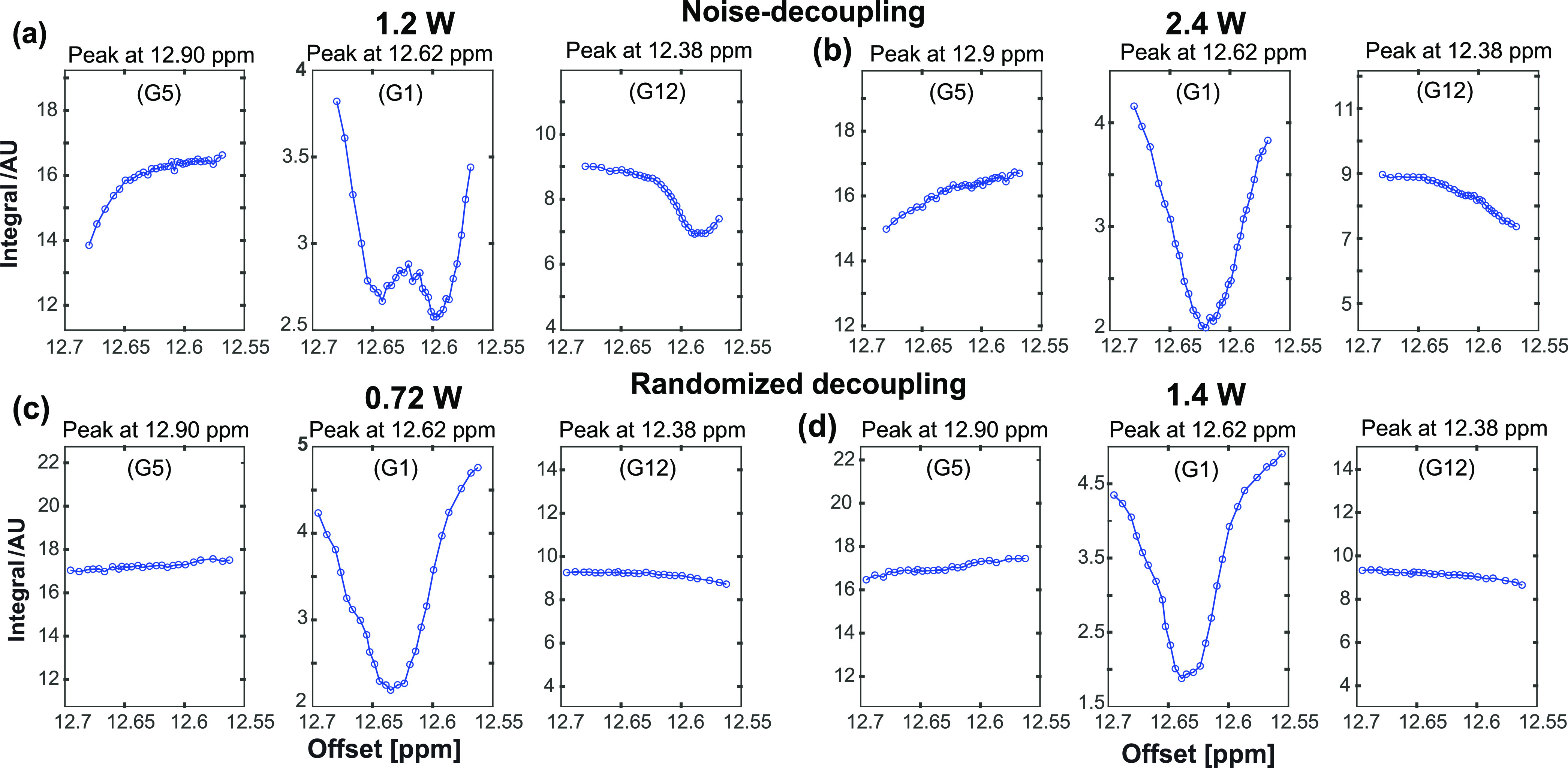
Offset-dependent saturation profiles for the saturation (800 ms, γB_1_/2π = 10 Hz) of G1 from the 14-mer RNA. Upper panels: random-noise ^15^N decoupling with (a) 1.2 W and (b) 2.4 W. Lower panels: randomized composite-pulse (90*_x_*–180*_y_*–90*_x_*) decoupling with (c) = 0.72 W and (d) = 1.4 W average decoupling powers, respectively.

Most decoupling schemes like WALTZ-16 or GARP-4^15^ rely on cyclic pulse trains that yield large effective decoupling bandwidths with moderate RF powers, but have sidebands as one of their intrinsic properties.^16^ Further improvements to these schemes like supercycling^17^ will, in general, increase the number of sidebands and bring them closer to the center peak, even if minimizing their intensities. This brings decoupling sidebands into the noise level of most NMR acquisitions, yet not of saturation-transfer experiments of the kind being considered here. Increasing the decoupling power will push these sidebands further out but not out of the spectral range of interest, particularly when involving the decoupling of ^15^N, a low-γ nucleus with relatively weak nutation fields. Similar effects were discussed in connection to ^2^H-decoupled low-power CEST experiments on ^13^C-labeled samples;^12,13^ to overcome these issues, Kalodimos et al. proposed ramped decoupling waveforms that “smear” C–D decoupling sidebands into the noise.^12^ Alternatively, a broader-band approach suitable for high-field ^15^N decoupling arises from applying random RF noise waveforms.^18,19^ Although this form of decoupling is insufficient to achieve sharp saturation profiles or even complete sideband suppression if implemented with 1.2 W (Figure 9a), 2.4 W will suffice to alleviate these limitations (Figure 9b). With a bandwidth ≤1 kHz, this will barely suffice to cover the ^15^N spectral widths of RNA (≈20 ppm) or protein backbone (≈30 ppm) imino protons at 11.7 T. To reinstate the efficiency benefits brought about by the composite pulse [90*_x_*–180*_y_*–90*_x_*] decoupling block while maintaining the sideband suppression performance of noise decoupling, a sequence that combined both approaches was assayed. In the ensuing “composite noise decoupling” scheme, the [90*_x_*–180*_y_*–90*_x_*] decoupling blocks were continuously concatenated, but the durations of these basic blocks were varied by pseudorandom alterations of their τ_90_(^15^N) pulse duration. For each [90*_x_*–180*_y_*–90*_x_*] block, the τ_90_(^15^N) was varied by up to 500% of the mean τ_90_(^15^N), and the RF power used in each block was adapted accordingly to ensure proper operation. Artifact-free spectra were thus obtained with ∼4× less RF power than in the conventional noise decoupling case (Figure 9c,d), providing sufficient bandwidths to decouple ^15^N spectral widths >30 ppm at 500 MHz, with 2.4 W on average.

## 3 Conclusions

While versatile, the use of selective excitation/inversion/saturation experiments for measuring cross-relaxation effects in biomolecules is not without its perils. Three confounding sources that may lead to false apparent cross-relaxation effects and/or suppress genuine ones were discussed here. The former included the “spill-over” of the saturation, and a self-saturation of the main resonances by irradiation of their otherwise invisible heteronuclear decoupling sidebands. The latter included “oversaturation” effects. The present study exemplified these different artifacts in relatively small-sized nucleic acids, where their presence was easier to appreciate. Peak crowding, however, is likely to make these confounding factors even more prevalent and even harder to recognize, as the size of the targeted molecules increases. In such instances, adding an additional dimension to the saturation experiments – for instance by including ^15^N resonances^8^ – would have been needed for achieving a clearer spectral characterization.

For the oligomers studied here, spill-over effects could be adequately identified by exploring the behavior of the spectra upon addressing frequencies adjacent to the main saturated resonance, while oversaturation can be avoided by reducing the saturating RF field as per eq 5. The magnifying effects that selective saturation could have on decoupling sidebands had been previously noted in CEST experiments;^12,13^ they can also involve ^1^H-decoupling and the observation of ^15^N or ^13^C resonances,^20^ but this tends to be less of a problem as ^1^H composite pulse decoupling pulses with short, τ_90_(^1^H) ≤ 50 μs durations are standard in NMR. By contrast, the short τ_90_(^15^N) pulses that would be required for providing similar bandwidths to ^1^H-based measurements are not usually available. This decoupling issue was thus solved here by the introduction of randomized approaches; it is likely that all of the issues hereby highlighted and addressed can also be solved by more sophisticated approaches, involving, for example, optimal control.

## 4 Experimental Section

### 4.1 Sample Preparation

The^15^N/^13^C-labeled CUUG-14-mer RNA sample was produced by T7 polymerase-based *in vitro* transcription, as described earlier.^21−23^ The final concentration of the RNA sample in the NMR tube was 1 mM. The unlabeled Dickerson-dodecamer DNA sample was purchased from Merck (Israel). The final concentration of the DNA sample in the NMR tube was 750 μM.

### 4.2 NMR Experiments

NMR experiments were conducted using a 500 MHz, 11.7 T Bruker Avance Neo spectrometer equipped with a Bruker Prodigy probe. All SMT experiments were performed by following the previously described procedure^7^ but without subtraction of an off-resonance spectrum to yield the saturation profiles. The duration of the saturation pulse was set to 800 ms with 10 Hz γB_1_/2π fields to saturate all imino resonances if not stated otherwise. Each spectrum was apodized with a QSINE window function and Fourier transformed using Topspin software (Bruker Biospin). Spectra were processed directly in Bruker TopSpin 4.1.1. To implement the decoupling schemes with randomized power levels/pulse lengths a text file was created manually and set up as a list for cpd. A script to create such pulse lengths in a more user-friendly fashion using Bruker’s au language has also been written and can be downloaded (at the user’s responsibility) from https://www.weizmann.ac.il/chembiophys/Frydman_group/software.

## Supporting Information Available

Numerical calculations confirming the validity of the analytical approximations employed here in the analysis of SMT NMR experiments (PDF)

## Notes

E̅.K. is an employee of the Bruker Corporation.

## Abbreviations

CEST: chemical exchange saturation transfer
NOE: nuclear Overhauser effect
NOESY: NOE spectroscopy
L- PROSY: loop-projected spectroscopy
HMT: Hadamard magnetization transfer
IDP: intrinsically disordered protein
RF: radiofrequency
SMT: selective magnetization transfer

## References

[ref1] Mayer M.; Meyer B. Characterization of Ligand Binding by Saturation Transfer Difference NMR Spectroscopy. Angew. Chem., Int. Ed. 1999, 38, 1784–1788. 10.1002/(sici)1521-3773(19990614)38:123.0.co;2-q. 29711196

[ref2] Vallurupalli P.; Bouvignies G.; Kay L. E. Studying “Invisible” Excited Protein States in Slow Exchange with a Major State Conformation. J. Am. Chem. Soc. 2012, 134, 8148–8161. 10.1021/ja3001419. 22554188

[ref3] Vallurupalli P.; Sekhar A.; Yuwen T.; Kay L. E. Probing Conformational Dynamics in Biomolecules via Chemical Exchange Saturation Transfer: A Primer.. J. Biomol. NMR 2017, 67, 243–271. 10.1007/s10858-017-0099-4. 28317074

[ref4] Rangadurai A.; Shi H.; Al-Hashimi H. M. Extending the Sensitivity of CEST NMR Spectroscopy to Micro-to-Millisecond Dynamics in Nucleic Acids Using High-Power Radio-Frequency Fields. Angew. Chem., Int. Ed. 2020, 59, 11262–11266. 10.1002/anie.202000493. PMC7857695 32168407

[ref5] Novakovic M.; Cousin S. F.; Jaroszewicz M. J.; Rosenzweig R.; Frydman L. Looped-PROjected SpectroscopY (L-PROSY): A Simple Approach to Enhance Backbone/Sidechain Cross-Peaks in 1H NMR. J. Magn. Reson. 2018, 294, 169–180. 10.1016/j.jmr.2018.07.010. 30064051

[ref6] Novakovic M.; Kupče E̅.; Oxenfarth A.; Battistel M. D.; Freedberg D. I.; Schwalbe H.; Frydman L. Sensitivity Enhancement of Homonuclear Multidimensional NMR Correlations for Labile Sites in Proteins, Polysaccharides, and Nucleic Acids. Nat. Commun. 2020, 11, 5317 10.1038/s41467-020-19108-x. 33087707 PMC7577996

[ref7] Novakovic M.; Kupče E̅.; Scherf T.; Oxenfarth A.; Schnieders R.; Grün J. T.; Wirmer-Bartoschek J.; Richter C.; Schwalbe H.; Frydman L. Magnetization Transfer to Enhance NOE Cross-Peaks among Labile Protons: Applications to Imino–Imino Sequential Walks in SARS-CoV-2-Derived RNAs. Angew. Chem., Int. Ed. 2021, 60, 11884–11891. 10.1002/anie.202015948. PMC8251384 33683819

[ref8] Kim J.; Novakovic M.; Jayanthi S.; Lupulescu A.; Kupce E.; Grün J. T.; Mertinkus K.; Oxenfarth A.; Richter C.; Schnieders R.; Wirmer-Bartoschek J.; Schwalbe H.; Frydman L. 3D Heteronuclear Magnetization Transfers for the Establishment of Secondary Structures in SARS-CoV-2-Derived RNAs. J. Am. Chem. Soc. 2021, 143, 4942–4948. 10.1021/jacs.1c01914. 33783202 PMC8154514

[ref9] Álvarez G. A.; Rao D. D. B.; Frydman L.; Kurizki G. Zeno and Anti-Zeno Polarization Control of Spin Ensembles by Induced Dephasing. Phys. Rev. Lett. 2010, 105, 160401 10.1103/PhysRevLett.105.160401. 21230950

[ref10] Bretschneider C. O.; Alvarez G. A.; Kurizki G.; Frydman L. Controlling Spin-Spin Network Dynamics by Repeated Projective Measurements. Phys. Rev. Lett. 2012, 108, 140403 10.1103/PhysRevLett.108.140403. 22540774 PMC5040494

[ref11] Ravikumar M.; Shukla R.; Bothner-By A. A. Relaxation and Dynamics of Coupled Spin Systems Subjected to Continuous Radio Frequency Fields. J. Chem. Phys. 1991, 95, 3092 10.1063/1.460866.

[ref12] Xia Y.; Yuwen T.; Liu A.; Kalodimos C. G. Removal of 2H-Decoupling Sidebands in 13CHD2 13C-CEST Profiles. J. Biomol. NMR 2021, 75, 133–142. 10.1007/s10858-021-00362-0. 33745068 PMC8342043

[ref13] Rennella E.; Huang R.; Velyvis A.; Kay L. E. 13CHD2–CEST NMR Spectroscopy Provides an Avenue for Studies of Conformational Exchange in High Molecular Weight Proteins. J. Biomol. NMR 2015, 63, 187–199. 10.1007/s10858-015-9974-z. 26271302

[ref14] Shaka A. J.; Keeler J. Broadband Spin Decoupling in Isotropic-Liquids. Prog. Nucl. Magn. Reson. Spectrosc. 1987, 19, 47–129. 10.1016/0079-6565(87)80008-0.

[ref15] Shaka A. J.; Keeler J.; Frenkiel T.; Freeman R. An Improved Sequence for Broadband Decoupling: WALTZ-16. J. Magn. Reson. 1983, 52, 335–338. 10.1016/0022-2364(83)90207-X.

[ref16] Shaka A. J.; Barker P. B.; Bauer C. J.; Freeman R. Cycling Sidebands in Broadband Decoupling. J. Magn. Reson. 1986, 67, 396–401. 10.1016/0022-2364(86)90451-8.

[ref17] Levitt M. H.; Freeman R.; Frenkiel T. Supercycles for Broadband Heteronuclear Decoupling. J. Magn. Reson. 1982, 50, 157–160. 10.1016/0022-2364(82)90042-7.

[ref18] Ernst R. R. Nuclear Magnetic Double Resonance with an Incoherent Radio-Frequency Field. J. Chem. Phys. 1966, 45, 3845–3861. 10.1063/1.1727409.

[ref19] Ernst R. R. NMR Studies of 19F Chemical Shifts and Coupling Constants in Cyclobutane Derivatives. Mol. Phys. 1969, 16, 241–255. 10.1080/00268976900100301.

[ref20] Chakrabarti K. S.; Ban D.; Pratihar S.; Reddy J. G.; Becker S.; Griesinger C.; Lee D. High-Power 1H Composite Pulse Decoupling Provides Artifact Free Exchange-Mediated Saturation Transfer (EST) Experiments. J. Magn. Reson. 2016, 269, 65–69. 10.1016/j.jmr.2016.05.013. 27240144

[ref21] Fürtig B.; Richter C.; Wöhnert J.; Schwalbe H. NMR Spectroscopy of RNA. ChemBioChem 2003, 4, 936–962. 10.1002/cbic.200300700. 14523911

[ref22] Schnieders R.; Knezic B.; Zetzsche H.; Sudakov A.; Matzel T.; Richter C.; Hengesbach M.; Schwalbe H.; Fürtig B. NMR Spectroscopy of Large Functional RNAs: From Sample Preparation to Low-Gamma Detection. Curr. Protoc. Nucleic Acid Chem. 2020, 82, e116 10.1002/cpnc.116. 32960489

[ref23] Wacker A.; Weigand J. E.; Akabayov S. R.; Altincekic N.; Bains J. K.; Banijamali E.; Binas O.; Castillo-Martinez J.; Cetiner E.; Ceylan B.; Chiu L.-Y.; Davila-Calderon J.; Dhamotharan K.; Duchardt-Ferner E.; Ferner J.; Frydman L.; Fürtig B.; Gallego J.; Grün J. T.; Hacker C.; Haddad C.; Hähnke M.; Hengesbach M.; Hiller F.; Hohmann K. F.; Hymon D.; de Jesus V.; Jonker H.; Keller H.; Knezic B.; Landgraf T.; Löhr F.; Luo L.; Mertinkus K. R.; Muhs C.; Novakovic M.; Oxenfarth A.; Palomino-Schätzlein M.; Petzold K.; Peter S. A.; Pyper D. J.; Qureshi N. S.; Riad M.; Richter C.; Saxena K.; Schamber T.; Scherf T.; Schlagnitweit J.; Schlundt A.; Schnieders R.; Schwalbe H.; Simba-Lahuasi A.; Sreeramulu S.; Stirnal E.; Sudakov A.; Tants J.-N.; Tolbert B. S.; Vögele J.; Weiß L.; Wirmer-Bartoschek J.; Martin M. A. W.; Wöhnert J.; Zetzsche H. Secondary Structure Determination of Conserved SARS-CoV-2 RNA Elements by NMR Spectroscopy. Nucleic Acids Res. 2020, 48, 12415–12435. 10.1093/nar/gkaa1013. 33167030 PMC7736788

