# Supplementary material for: Identifying and Overcoming Artifacts in 1 H-Based Saturation Transfer NOE NMR Experiments
Source: J Am Chem Soc. 2023 Mar 6;145(11):6289–98. doi: 10.1021/jacs.2c13087 (PMC10037324; doi:10.1021/jacs.2c13087)
Supplement: Supplementary file 1 — ja2c13087_si_001.pdf [file ja2c13087_si_001.pdf]

SUPPORTING INFORMATION

Identifying and Overcoming Artifacts in ^{1}H-based Saturation Transfer NOE NMR Experiments

J. Tassilo Grün[1], Jihyun Kim[1], Sundaresan Jayanthi[2], Adonis Lupulescu[3], Ēriks Kupče[4], Harald Schwalbe[5], and Lucio Frydman[1*]

[1]Department of Chemical and Biological Physics, Weizmann Institute of Science, Rehovot 7610001, Israel

[2]Department of Physics, Indian Institute of Space Science and Technology, Valiamala, 695547 Thiruvananthapuram, Kerala, India

[3]Extreme Light Infrastructure—Nuclear Physics ELI-NP, Laser Gamma Experiments Department (LGED), "Horia Hulubei" National Institute for Physics and Nuclear Engineering IFIN-HH, 30 Reactorului Street, 077125 Bucharest-Măgurele, Romania

[4]Bruker Ltd, Banner Lane, Coventry, CV4 9TT, UK

[5]Institute for Organic Chemistry and Chemical Biology, Center for Biomolecular Magnetic Resonance, Goethe-University, 60438 Frankfurt/Main, Germany

On the validity of the analytical approximations to saturation-transfer's numerical solutions

Figure S1 compares plots of Z_A and Z_B values derived from numerical solutions of Eq. [1] for long irradiation times and a variety of irradiation offsets (points), against the offset-dependent curves predicted for Z_A and Z_B by the analytical expressions in Eqs. [2] and [3] of the main text. These data display excellent agreement for $\sigma_t = 0$, and small deviations in Z_B only when $\sigma_t \neq 0$. However, even in such cases, when the magnetization is calculated at the $\delta = 0$ point that will be of main interest in SMT, the maximum deviation in $Z_B(\delta = 0)$ is ≤ 0.3%. Therefore, working without σ_t is reasonably justified.

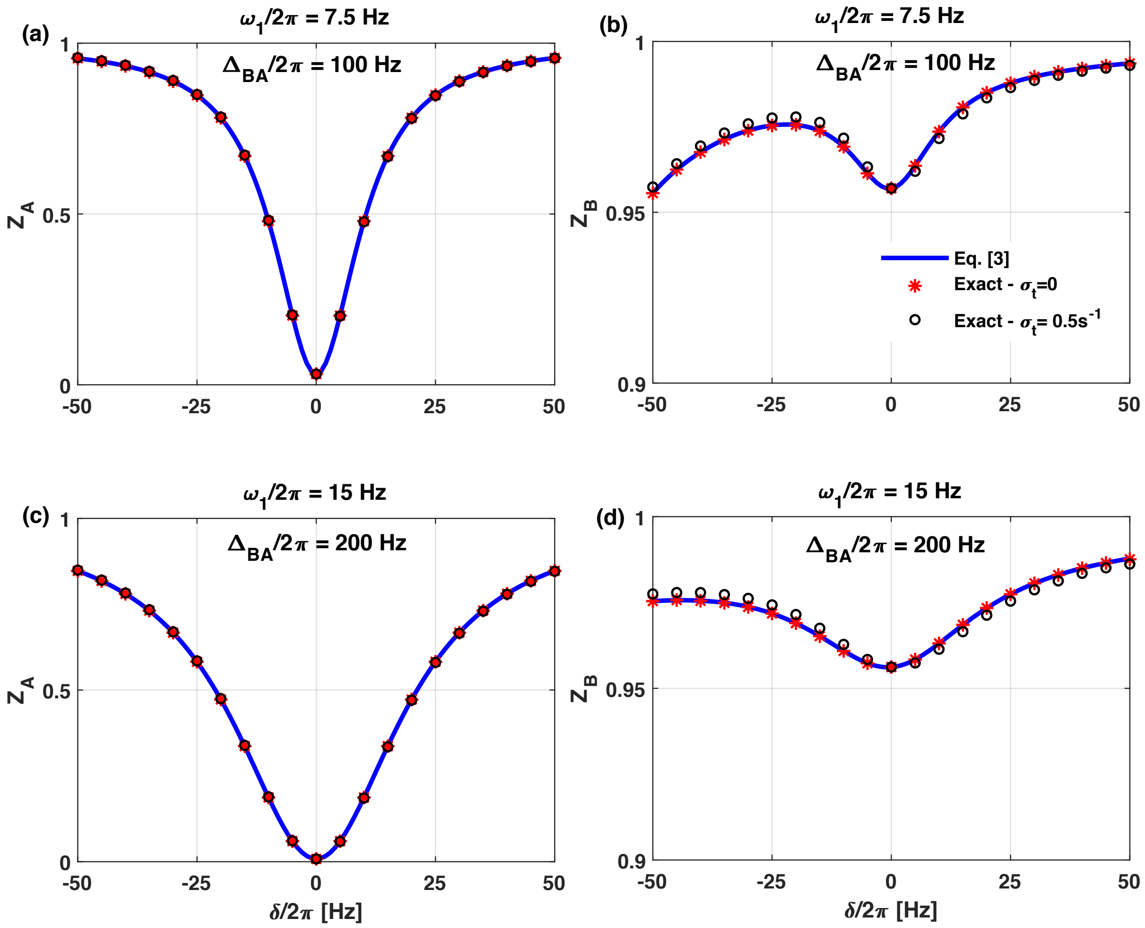

Figure S1. Z_A, Z_B plotted as function of $\delta/2\pi$ off-resonance irradiation offset from ω_A=0, for parameters $\sigma_l = -0.2\ \mathrm{s}^{-1}$, $\rho_t = 12\ s^{-1}$, $\rho_l = 6\ \mathrm{s}^{-1}$ –with and without considering $\sigma_t = 0.5\ \mathrm{s}^{-1}$ Chemical shift differences were **(a, b)** $\Delta_{BA}/2\pi = 100$ Hz and ($\mathbf{c}, \mathbf{d}$)200 Hz. Assumed RF amplitudes were $\omega_1/2\pi = 7.5$ Hz **(a,b)** and $\omega_1/2\pi = 15$ Hz **(c,d)**. The values $\sigma_l = -0.2\ \mathrm{s}^{-1}, \sigma_t = 0.5\ \mathrm{s}^{-1}$ correspond to an internuclear distance of 2.5 Å, a correlation time ~0.9 ns, and a proton Larmor frequency of 500 MHz.

Eqs. (2) and (3) can be used to derive general expressions for the second derivatives of the saturation curves. In particular the $\frac{d^2 Z_B}{d\delta^2}$ defining the visibility of a cross-relaxation A→B dip can be written, for any irradiation offset δ, as

$$\frac{d^2 Z_B}{d\delta^2} = 4\pi^2 \sum_{i=1}^{9} T_i \qquad [S1]$$

where

$$T_1 = \frac{2\rho_t\omega_1^2(\rho_l+\sigma_l)}{((\omega_A+\delta)^2+\rho_t^2)^2\left(\sigma_l^2-\left(\rho_l+\frac{\rho_t\omega_1^2}{(\omega_A+\delta)^2+\rho_t^2}\right)\left(\rho_l+\frac{\rho_t\omega_1^2}{(\omega_B+\delta)^2+\rho_t^2}\right)\right)}$$

$$T_2 = -\frac{8\rho_t\omega_1^2(\omega_A+\delta)^2(\rho_l+\sigma_l)\left(\rho_l-\sigma_l+\frac{\rho_t\omega_1^2}{((\omega_A+\delta)^2+\rho_{t2})}\right)\left(\rho_l+\frac{\rho_t\omega_1^2}{(\omega_B+\delta)^2+\rho_t^2}\right)}{\left(\sigma_l^2-\left(\rho_l+\frac{\rho_t\omega_1^2}{(\omega_A+\delta)^2+\rho_t^2}\right)\left(\rho_l+\frac{\rho_t\omega_1^2}{(\omega_B+\delta)^2+\rho_t^2}\right)\right)^2((\omega_A+\delta)^2+\rho_t^2)^3}$$

$$T_3 = \frac{2\rho_t\omega_1^2\left(\rho_l+\frac{\rho_t\omega_1^2}{(\omega_B+\delta)^2+\rho_t^2}\right)(\rho_l+\sigma_l)\left(\rho_l-\sigma_l+\frac{\rho_t\omega_1^2}{((\omega_A+\delta)^2+\rho_{t2})}\right)}{((\omega_A+\delta)^2+\rho_t^2)^2\left(\sigma_l^2-\left(\rho_l+\frac{\rho_t\omega_1^2}{(\omega_A+\delta)^2+\rho_t^2}\right)\left(\rho_l+\frac{\rho_t\omega_1^2}{(\omega_B+\delta)^2+\rho_t^2}\right)\right)^2}$$

$$T_4 = \frac{2\rho_t\omega_1^2\left(\rho_l+\frac{\rho_t\omega_1^2}{((\omega_A+\delta)^2+\rho_{t2})}\right)(\rho_l+\sigma_l)\left(\rho_l-\sigma_l+\frac{\rho_t\omega_1^2}{((\omega_A+\delta)^2+\rho_{t2})}\right)}{((\omega_B+\delta)^2+\rho_t^2)^2\left(\sigma_l^2-\left(\rho_l+\frac{\rho_t\omega_1^2}{(\omega_A+\delta)^2+\rho_t^2}\right)\left(\rho_l+\frac{\rho_t\omega_1^2}{(\omega_B+\delta)^2+\rho_t^2}\right)\right)^2}$$

$$T_5 = -\frac{8\rho_t\omega_1^2\left(\rho_l+\frac{\rho_t\omega_1^2}{((\omega_A+\delta)^2+\rho_{t2})}\right)(\omega_B+\delta)^2}{((\omega_B+\delta)^2+\rho_t^2)^3\left(\sigma_l^2-\left(\rho_l+\frac{\rho_t\omega_1^2}{(\omega_A+\delta)^2+\rho_t^2}\right)\left(\rho_l+\frac{\rho_t\omega_1^2}{(\omega_B+\delta)^2+\rho_t^2}\right)\right)^2}$$

$$T_6 = -\frac{8\rho_t^2\omega_1^4(\omega_B+\delta)(\omega_A+\delta)}{(\omega_A+\delta)^2(\omega_B+\delta)^2\left(\sigma_l^2-\left(\rho_l+\frac{\rho_t\omega_1^2}{(\omega_A+\delta)^2+\rho_t^2}\right)\left(\rho_l+\frac{\rho_t\omega_1^2}{(\omega_B+\delta)^2+\rho_t^2}\right)\right)^2}$$

$$T_7 = -\frac{8\rho_t^2\omega_1^4(\rho_l+\sigma_l)\left(\rho_l-\sigma_l+\frac{\rho_t\omega_1^2}{((\omega_A+\delta)^2+\rho_{t2})}\right)\left(\frac{\left(\rho_l+\frac{\rho_t\omega_1^2}{(\omega_B+\delta)^2+\rho_t^2}\right)(\omega_A+\delta)}{((\omega_A+\delta)^2+\rho_t^2)^2}+\frac{(\omega_B+\delta)\left(\rho_l+\frac{\rho_t\omega_1^2}{((\omega_A+\delta)^2+\rho_{t2})}\right)}{((\omega_A+\delta)^2+\rho_t^2)^2}\right)^2}{\left(\sigma_l^2-\left(\rho_l+\frac{\rho_t\omega_1^2}{(\omega_A+\delta)^2+\rho_t^2}\right)\left(\rho_l+\frac{\rho_t\omega_1^2}{(\omega_B+\delta)^2+\rho_t^2}\right)\right)^3}$$

$$T_8 = -\frac{8\rho_t\omega_1^2(\rho_l+\sigma_l)(\omega_A+\delta)^2}{\left(\sigma_l^2-\left(\rho_l+\frac{\rho_t\omega_1^2}{(\omega_A+\delta)^2+\rho_t^2}\right)\left(\rho_l+\frac{\rho_t\omega_1^2}{(\omega_B+\delta)^2+\rho_t^2}\right)\right)((\omega_A+\delta)^2+\rho_t^2)^3}$$

$$T_9 = -\frac{4\rho_t\omega_1^2(\rho_t + \sigma_l)(\omega_A + \delta)\left(\frac{\left(\rho_l + \frac{\rho_t\omega_1^2}{(\omega_B+\delta)^2+\rho_t^2}\right)(\omega_A+\delta)}{((\omega_A+\delta)^2+\rho_t^2)^2} + \frac{(\omega_B+\delta)\left(\rho_l + \frac{\rho_t\omega_1^2}{((\omega_A+\delta)^2+\rho_{t2})}\right)}{((\omega_A+\delta)^2+\rho_t^2)^2}\right)}{\left(\sigma_l^2 - \left(\rho_l + \frac{\rho_t\omega_1^2}{(\omega_A+\delta)^2+\rho_t^2}\right)\left(\rho_l + \frac{\rho_t\omega_1^2}{(\omega_B+\delta)^2+\rho_t^2}\right)\right)^2((\omega_A + \delta)^2 + \rho_t^2)^2}$$

At $\delta = 0$, this expression for $\frac{d^2 Z_B}{d\delta^2}$ reduces to,

$$\left(\frac{d^2 Z_B}{d\delta^2}\right)_{\delta=0} = 4\pi^2 \sum_{i=1}^{5} t_i \qquad \text{[S2]}$$

where

$$t_1 = \frac{2\omega_1^2(\rho_l + \sigma_l)\left(\frac{\omega_1^2}{\rho_t} + \rho_l - \sigma_l\right)}{\rho_t^3\left(\rho_l + \frac{\omega_1^2}{\rho_t}\right)^2\left(\rho_l + \frac{\omega_1^2\rho_t}{\rho_t^2 + \Delta_{BA}^2}\right)}$$

$$t_2 = \frac{2\rho_t\omega_1^2(\rho_l + \sigma_l)\left(\frac{\omega_1^2}{\rho_t} + \rho_l - \sigma_l\right)}{(\rho_t^2 + \Delta_{BA}^2)^2\left(\rho_l + \frac{\omega_1^2}{\rho_t}\right)\left(\rho_l + \frac{\omega_1^2\rho_t}{\rho_t^2 + \Delta_{BA}^2}\right)^2}$$

$$t_3 = \frac{-8\rho_t\omega_1^2\Delta_{BA}^2(\rho_l + \sigma_l)\left(\frac{\omega_1^2}{\rho_t} + \rho_l - \sigma_l\right)}{(\rho_t^2 + \Delta_{BA}^2)^3\left(\rho_l + \frac{\omega_1^2}{\rho_t}\right)\left(\rho_l + \frac{\omega_1^2\rho_t}{\rho_t^2 + \Delta_{BA}^2}\right)^2}$$

$$t_4 = \frac{-2\omega_1^2(\rho_l + \sigma_l)}{\rho_t^3\left(\rho_l + \frac{\omega_1^2}{\rho_t}\right)\left(\rho_l + \frac{\omega_1^2\rho_t}{\rho_t^2 + \Delta_{BA}^2}\right)}$$

$$t_5 = \frac{8\rho_t^2\omega_1^4\Delta_{BA}^2(\rho_l + \sigma_l)\left(\frac{\omega_1^2}{\rho_t} + \rho_l - \sigma_l\right)}{(\rho_t^2 + \Delta_{BA}^2)^4\left(\rho_l + \frac{\omega_1^2}{\rho_t}\right)\left(\rho_l + \frac{\omega_1^2\rho_t}{\rho_t^2 + \Delta_{BA}^2}\right)^3}$$

While these are not friendly expressions, Eq. [S2] can be considerably simplified if ω_1^2 is significantly larger than $\rho_t\rho_l$. As mentioned in the main text, this leads to

$$\left(\frac{d^2 Z_B}{d\delta^2}\right)_{\delta=0} \cong \left(-\frac{2\sigma_l}{\rho_t\omega_1^2} - \frac{6\rho_t\omega_1^2}{\Delta_{BA}^4\rho_l}\right) \qquad \text{[S3]}$$

As the first term in Eq. [S3] is proportional to $1/\omega_1^2$, this approximate expression diverges when $\omega_1 \rightarrow 0$. While this is not the case for the more exact equation Eq. [S2], the extent to which these two will differ will depend on Δ_{BA}: the smaller the latter, the earlier the differences set in. Comparisons between the predictions of Eqs. [S2] and [S3] for different relaxation rates and chemical shift differences are shown in Figures S2 and S3. Also shown in Figure S4 are high RF saturation power regimes, where the simplifying assumptions underlying Eq. [S3] are broken. As can be seen, for all RF amplitudes which are of relevance to saturate the A resonance and hence lead to observable NOE effects, Eq. [S3] makes sounds predictions.

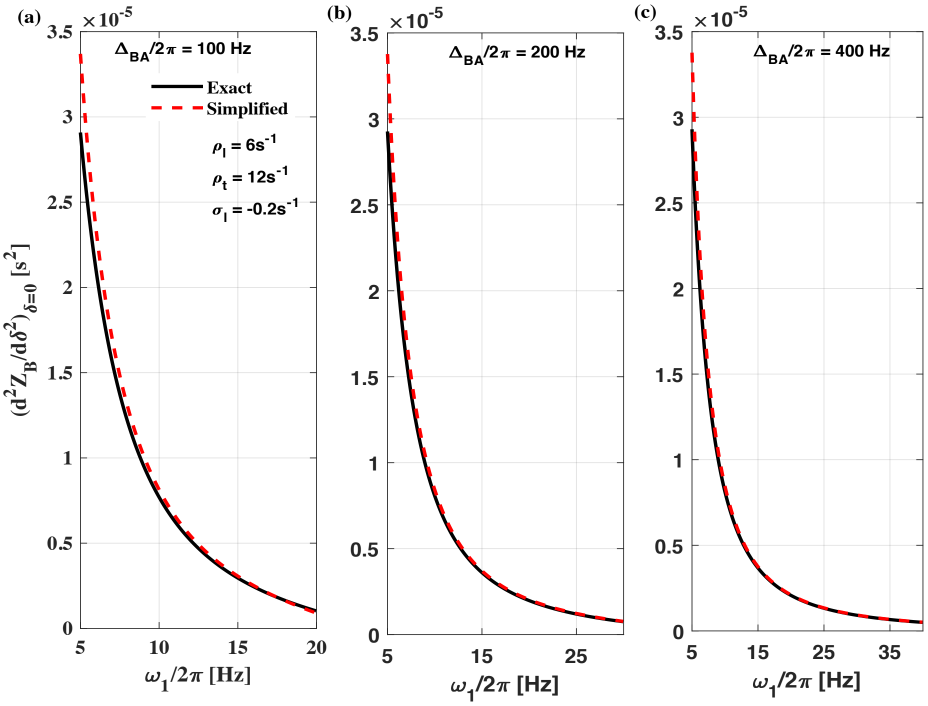

Figure S2. Comparative plots of Eq. [4] and Eq. [S1] for a range of RF saturation fields applied on-resonance on A. Relaxation rates are $\sigma_l = -0.2\ s^{-1}$, $\rho_t = 12\ s^{-1}$, $\rho_l = 6\ s^{-1}$

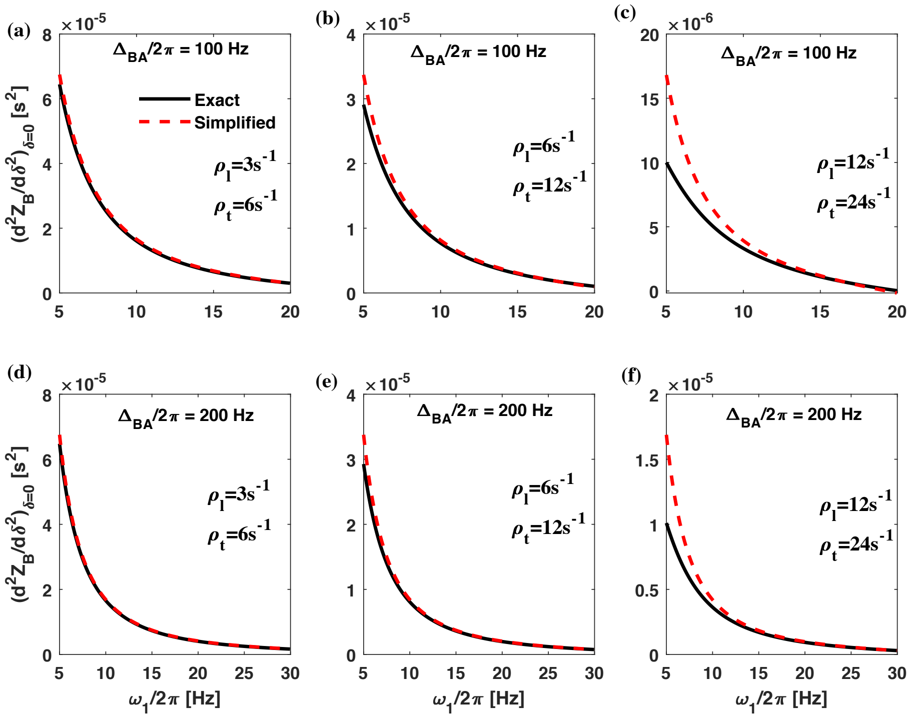

Figure S3. Idem as Figure S2 for two sites with chemical shift differences of 100 Hz **(a-c)**, and 200 Hz **(d-f)**, and for the wider range of relaxation parameters shown in the respective plots. $\sigma_l = -0.2\ s^{-1}$ in all simulations.

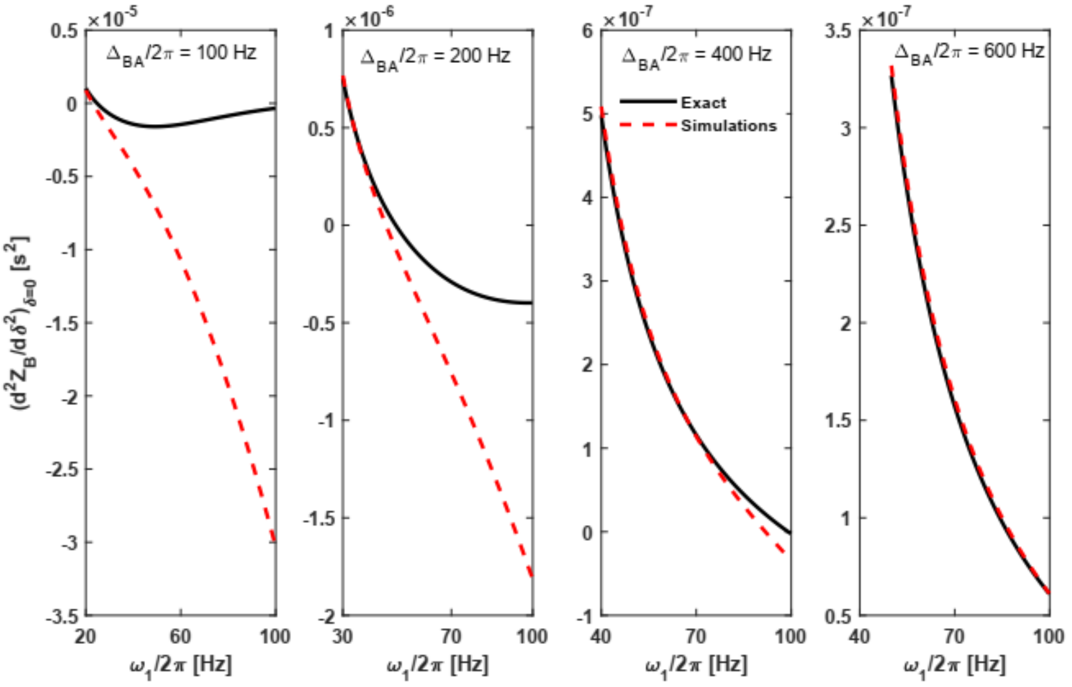

Figure S4. Comparative plots of Eqs. [S3] and the predictions stemming from Eq. [S1] for large RF values. Relaxation rates are $\sigma_l = -0.2s^{-1}$, $\rho_t = 12\ s^{-1}$, $\rho_l = 6\ s^{-1}$.
